# Diagnostic and Prognostic Value of a TDI-Derived Systolic Wall Motion Analysis as a Screening Modality for Allograft Rejection after Heart Transplantation

**DOI:** 10.3390/life11111206

**Published:** 2021-11-09

**Authors:** Isabell A. Just, Meryem Guelfirat, Laura Leser, Ata Uecertas, Laurenz Kopp Fernandes, Maren Godde, Nicolas Merke, Philipp Stawowy, Felix Hennig, Christoph Knosalla, Volkmar Falk, Jan Knierim, Felix Schoenrath

**Affiliations:** 1 Department of Cardiothoracic and Vascular Surgery, German Heart Center Berlin, 13353 Berlin, Germany; meryemguelfirat@gmail.com (M.G.); uecertas@dhzb.de (A.U.); kopp@dhzb.de (L.K.F.); godde@dhzb.de (M.G.); merke@dhzb.de (N.M.); hennig@dhzb.de (F.H.); knosalla@dhzb.de (C.K.); falk@dhzb.de (V.F.); knierim@dhzb.de (J.K.); schoenrath@dhzb.de (F.S.); 2 DZHK (German Centre for Cardiovascular Research), Partner Site Berlin, 10785 Berlin, Germany; stawowy@dhzb.de; 3 Department of Anesthesiology, German Heart Center Berlin, 13353 Berlin, Germany; leser@dhzb.de; 4 Department of Cardiology and Internal Medicine, German Heart Center Berlin, 13353 Berlin, Germany; 5 Department of Cardiothorarcic Surgery, Charité, Corpoate Member of Freie Universität Berlin, Humboldt-Universitüt Berlin and Berlin Institute of Health, 13353 Berlin, Germany; 6 Translational Cardiovascular Technologies, Department of Health Sciences, Eidgenoessische Technische Hochschule (ETH) Zurich, 8092 Zurich, Switzerland

**Keywords:** heart transplantation, rejection, surveillance, echocardiography

## Abstract

Background: Despite the risk for complications, allograft surveillance after orthotopic heart transplantation (OHT) is performed by cardiac catheterization and biopsies. We investigated the diagnostic and prognostic value of a TDI-derived systolic wall motion analysis of the posterobasal wall of the left ventricle (Sm) as a screening modality in OHT aftercare. Methods: We examined data of 210 eligible patients who underwent OHT between 2010 and 2020. Forty-four patients who had died within the initial hospital stay were excluded. For 166 patients, baseline and follow-up data were analyzed. The mean age at OHT was 46.2 (±11.4) years; 76.5% were male. Results: Within the observational period, 22 (13.3%) patients died. In total, 170 episodes of acute cellular or humoral rejections occurred (84 ISHLT1R; 13 ISHLT2R; 8 ISHLT3R; 65 AMR), and 29 catheterizations revealed cardiac allograft vasculopathy (5 CAV1; 4 CAV2; 20 CAV3). Individual Sm radial/longitudinal remained stable within the follow-up period (11.5 ± 2.2 cm/s; 10.9 ± 2.1 cm/s). Patients with acute rejections and CAV3 showed significant Sm radial/longitudinal reductions (AMR1: 1.6 ± 1.9 cm/s, confidence interval (CI) 0.77–0.243, *p* < 0.001; 1.8 ± 2.0 cm/s, CI 0.92–0.267, *p* < 0.001. ISHLT1R: 1.7 ± 1.8 cm/s, CI 1.32–2.08, *p* < 0.001; 2.0 ± 1.6 cm/s, CI 1.66–2.34, *p* < 0.001. CAV3: 1.3 ± 2.5 cm/s, CI 0.23–2.43, *p* < 0.017; 1.4 ± 2.8 cm/s, CI 0.21–2.66, *p* < 0.021). Lower Sm was associated with a threefold increase in all-cause mortality (hazard ratio (HR) 3.24, CI 1.2–8.76, *p* = 0.020; HR 2.92, CI 1.19–7.18, *p* = 0.019). Overall, Sm-triggered surveillance led to 0.75 invasive diagnostics per patient post-OHT year. Conclusions: Sm remained stable in the post-OHT course. Reductions indicated ISHLT1R, AMR1 and CAV3 and were associated with higher all-cause mortality. Sm-triggered surveillance may be referred to as a safe, high-yield screening modality in OHT aftercare.

## 1. Introduction

Acute rejection (AR) and cardiac allograft vasculopathy (CAV) are leading causes of mortality after orthotopic heart transplantation (OHT) [1]. Approximately one-quarter of patients experience at least one AR within the first year; in one-third of patients, CAV occurs within 5 years after OHT [2,3].

The early diagnosis and treatment of these complications are crucial in the follow-up care of heart transplant recipients. Since the symptoms and clinical signs of AR and CAV are often unspecific and typically occur late, routine surveillance by endomyocardial biopsies (EMB) and angiographies are recommended every six to twelve months if the renal function is not severely impaired [4]. Procedures are accompanied by a risk for acute complications and for the development of tricuspid valve regurgitation after repetitive catheter passages [5]. Furthermore, invasive follow-ups consume hospital resources and are of limited acceptance by the patients, leading caregivers to keep the frequency of invasive diagnostics as low as possible. However, this results in a period of uncertainty between examinations, which might be particularly challenging in patients with persisting donor-specific human leukocyte antigene (HLA) antibodies or false negative EMB. Furthermore, routine surveillances of asymptomatic patients may reveal clinically insignificant, low-grade rejections, and lead to immunosuppressant overtreatment with a concomitant risk for infections and malignancies [6].

Despite advances in noninvasive cardiac imaging, a reliable, easily accessible screening modality for follow-up care after OHT has not yet been widely introduced in clinical practice. In our institution, echocardiographic AR and CAV surveillance via pulsed-wave tissue Doppler imaging (PW-TDI) has been routinely performed and used for navigation of invasive diagnostics for more than 15 years.

The aim of this retrospective analysis was to investigate the long-term course, diagnostic reliability, and prognostic value of systolic wall motion analysis after OHT.

## 2. Materials and Methods

**Figure 1:**
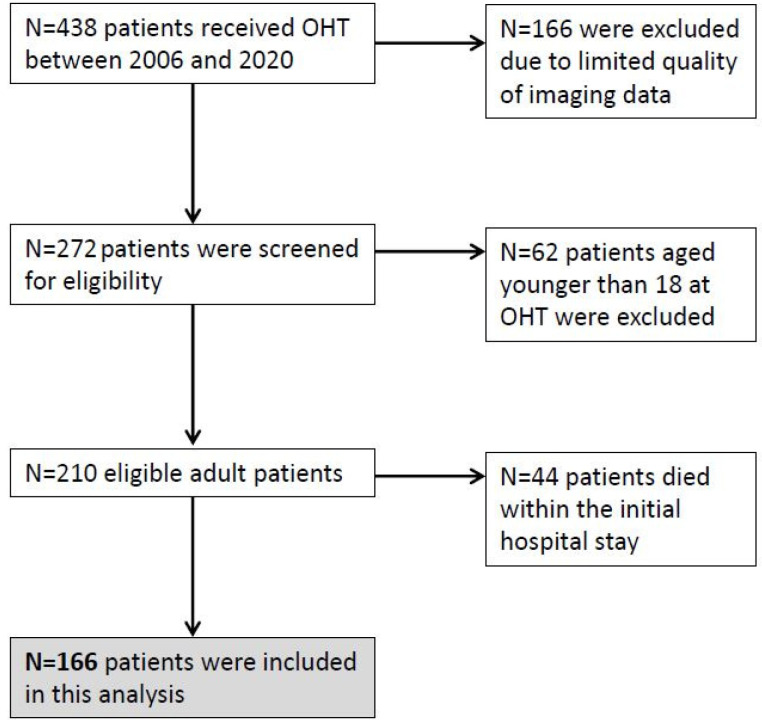
Study cohort.

The files of 438 patients who had undergone heart transplantation at our institution between 2006 and 2020 were retrospectively analyzed. Due to technical restrictions of the image data storage before 2010, only patients who had received a transplantation between 2010 and 2020 were further analyzed for eligibility. Recipients aged under 18 years at OHT and patients who had died within the transplant hospital stay were excluded from final analysis (Figure 1).

The baseline characteristics of 166 donors and recipients, including age, sex, body mass index, cause of heart failure, concomitant diseases; intra- and postoperative data, including ischemic time, immunosuppressive treatment, and intensive care unit stay; and echocardiographic follow-up data from more than 1100 visits were collected in an electronic database. The study conforms to the principles outlined in the Declaration of Helsinki and was approved by the local ethics committee (EA2/169/19).

AR is referred to as T-cell-mediated acute cellular rejection (ACR), antibody mediated rejection (AMR), or both. AR grading was defined according to the classification of The International Society for Heart and Lung Transplantation (ISHLT) [7,8]. CAV affects both epicardial and intramural coronary arteries, and is characterized by diffuse intimal proliferation with successive lumen narrowing and microvascular dysfunction [9,10]. The grading was defined by the degree of stenosis, number and localization of lesions, and graft function, as described by the ISHLT [11].

**Figure 2:**
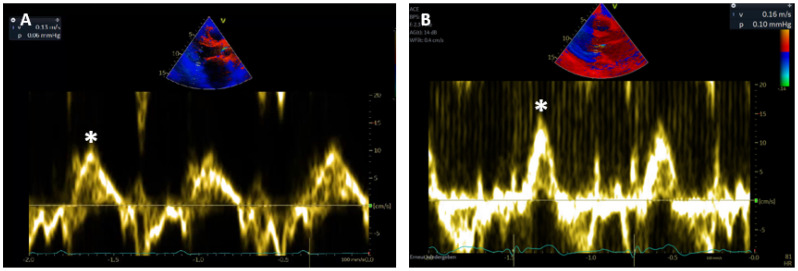
(**A**) Longitudinal and (**B**) radial Sm measured by PW-TDI at basal posterolateral segment of the LV (*).

Systolic wall motion peak velocities (Sm) were measured by PW-TDI at the subendocardial, posterobasal segment of the left ventricle at the level of the mitral leaflet tips. Radial wall motion was measured in parasternal long axis view and longitudinal wall motion in apical 3-chamber view [12]. In routine echocardiography, measurements of the Sm during or after extrasystoles are excluded from analysis. Since transducer angulation might result in a detection of lower velocities, the highest systolic wall motion velocity from at least three beats was selected in clinical routine (Figure 2).

Clinical, electrocardiographic, and echocardiographic signs of graft failure were routinely evaluated every three to six months in asymptomatic patients in OHT aftercare in our institution. If Sm values decreased by >10% compared to individual previous values, invasive diagnostics, including coronary angiography and biopsies, were performed.

Continuous data are summarized as mean and standard deviation (SD) or, in the case of skewed data, as median and interquartile range (IQR). Comparisons between groups were analyzed using t-tests for paired samples. Due to the small samples, t-tests were not calculated for the following groups: Sm reduction in ACR ISHLT 2R and 3Rm and ISHLT CAV1 and CAV2. Frequencies and percentages are reported for categorical data. The association of echocardiographic parameters with all-cause mortality was tested in a time-varying covariate Cox regression model. For patients who survived the first year after transplantation, conditional survival rates were analyzed using the Kaplan–Meier method. SPSS 25 and R 4.0 were used for statistical analysis.

## 3. Results

### 3.1. Patient Characteristics

Between 2010 and 2020, 166 adult patients received OHT and survived the transplant hospital stay. The mean age of recipients was 46.2 (±11.4) years at OHT; donors were aged 40.8 (±14.1) years. Overall, 127 (76.5%) recipients and 107 (64.5%) donors were male. Most patients (88.0%) underwent heart transplantation in a highly urgent status on the waiting list of Eurotransplant with terminal heart failure, mainly due to dilated cardiomyopathy (68.7%) or ischemic heart disease (21.1%). Half of the patients (50.6%) were on long-term mechanical circulatory support before OHT, and three (1.8%) patients received a combined transplantation of heart and kidney.

Mean ischemic time was 259.9 (±63.3) minutes. Immunosuppressive therapy included methylprednisolone, calcineurin inhibitors (97.4% cyclosporine, 2.4% tacrolimus), and antithymocyte globulin (60.4%) at induction and methylprednisolone, calcineurin inhibitors (55.4% cyclosporine, 42.8% tacrolimus) and mycophenolate mofetil (79.5%) at discharge. Mean hospital stay was 62.3(±50.7) days (Table 1).

### 3.2. Invasive Diagnostics in OHT Follow-Up

**Figure 3:**
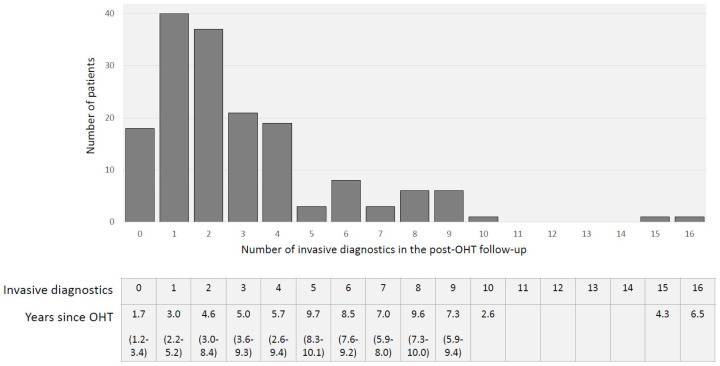
Total number of invasive diagnostics, including EMB and angiographies, during OHT follow-up and years since OHT (median (IQR)).

Overall, 480 invasive diagnostics, including 233 EMB, 38 angiographies, and 208 combined procedures, were performed, resulting in 0.75 invasive diagnostics per patient post-transplant year (Figure 3).

### 3.3. Mortality, AR and CAV

**Figure 4:**
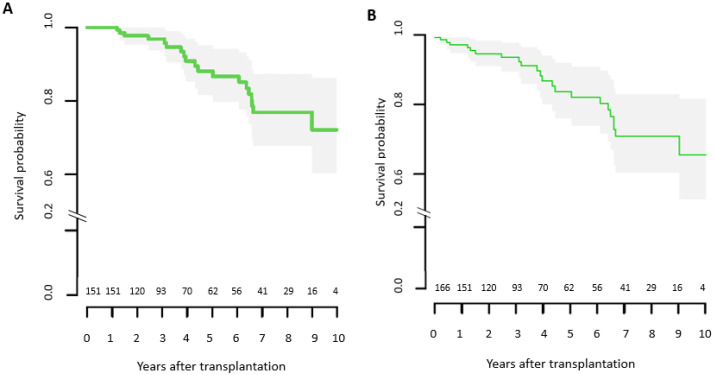
(**A**) Survival after OHT conditional on surviving to 1 year; (**B**) survival of study population.

Of all patients, 13.3% (*n* = 22) died in a mean of 4.1 (±2.4) years after OHT. Figure 4 presents the survival rates of patients who survived the first post-transplant year. Causes of death were graft failure due to AR and CAV in 6 (27.3%) and 4 (18.2%) patients, respectively. Five (22.7%) patients died due to an infection (necrotizing pancreatitis, mediastinitis, intracranial abscess, sepsis of other origins) and 2 (9.1%) patients due to lung cancer. In 4 (18.2%) patients, the cause of death was unknown.

In total, 170 episodes of AR (detected in 480 (34.5%) invasive procedures) occurred post OHT follow-up. Of these ARs, 84 (49.4%) were classified as mild ACR ISHLT1R, 13 (7.6%) as moderate ACR ISHLT2R, and 8 (4.7%) as severe ACR ISHLT3R; 65 (38.2%) were caused by AMR. In 29 cardiac catheterizations, CAV was diagnosed: 5 (17.2%) cases showed mild ISHLT CAV1, 4 (13.8%) cases moderate ISHLT CAV2, and 20 (69.0%) cases severe ISHLT CAV3. Of the 4 ISHLT CAV2 cases, 2 were progressions of a previously known CAV, and of the 20 ISHLT CAV3 cases, 8 were progressions of a previously known CAV.

### 3.4. Sm Course after OHT

**Figure 5:**
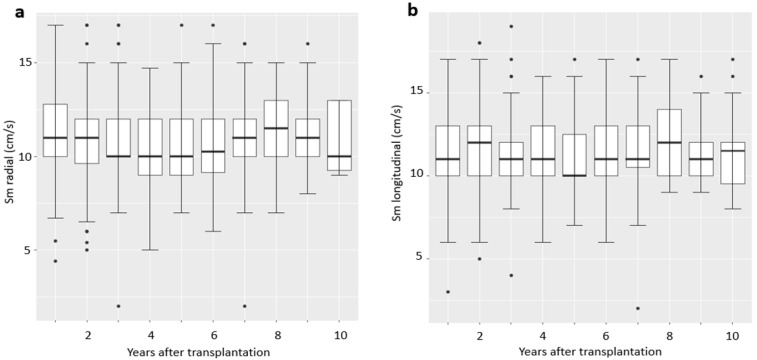
Course of (**a**) radial and (**b**) longitudinal Sm from discharge after OHT.

Individual Sm values remained stable from discharge after OHT over the follow-up period. Radial and longitudinal Sm were 11.5 ± 2.2 and 10.9 ± 2.1 cm/s, respectively (Figure 5).

### 3.5. Predictive Value of Sm

Patients with mild ACR ISHLT 1R or AR caused by AMR and ISHLT CAV3 showed intraindividual significant reductions in radial and longitudinal Sm with a decrease from 1.7 ± 1.8 cm/s and 2.0 ± 1.6 cm/s, CI 1.32–2.08, *p* < 0.001 and CI 1.66–2.34, *p* < 0.001, respectively in ACR ISHLT 1R; 1.6 ± 1.9 cm/s and 1.8 ± 2.0 cm/s, 95% CI 0.77–0.243, *p* < 0.001 and CI 0.92–0.267, *p* < 0.001, respectively in AMR; and 1.3 ± 2.5 cm/s and 1.4 ± 2.8 cm/s, CI 0.23–2.43, *p* < 0.017 and CI 0.21–2.66, *p* < 0.021, respectively in ISHLT CAV3 (Table 2 and Table 3).

### 3.6. Prognostic Value of Sm

In a time-varying covariate Cox regression model, lower radial or longitudinal Sm values at any time were associated with higher all-cause mortality per cm/s decrease (hazard ratio (HR) 0.77, CI 0.63–0.93, *p* = 0.008; HR 0.7, CI 0.58–0.95, *p* = 0.016; HR, respectively), as presented in Table 4. A radial Sm of less than 9 cm/s and a longitudinal Sm of less than 10 cm/s were associated with an approximately threefold increased risk of mortality (HR 3.24, CI 1.2–8.76, *p* = 0.020; HR 2.92, CI 1.19–7.18, *p* = 0.019; HR, respectively).

## 4. Discussion

Our analysis shows that the PW-TDI-derived systolic wall motion of the posterobasal segment of the left ventricle remains stable for at least 10 years after OHT. A decrease in Sm may indicate AR or CAV and is a predictor of mortality.

After Dandel et al. described the high diagnostic value of Sm measurements for predicting ACR and CAV, and its usefulness for the scheduling of invasive diagnostics in a prospective clinical trial in 2001, our institutional routine post-transplant care was adapted with a shift from routine surveillance to a clinical–echocardiographic navigation of EMB and angiography [12,13,14]. The present work represents a long-term follow-up of our experiences following this novel approach. In our recent analysis, low-grade ACR and high-grade CAV were accompanied by a significant reduction in Sm. This complements previously published work, where medium- and high-grade ACR (ISHLT ≥ 2R), and low-grade or angiographically invisible CAV were reliably indicated by a Sm decrease [12,13,14]. Additionally, we first describe here a significant Sm reduction in patients with AR caused by AMR (which was defined by the ISHLT in 2005) [7].

Moreover, in our analysis, we observed a stable long-term Sm course, which supports the usefulness of Sm surveillance over the post-OHT period. A one-time detection of radial or longitudinal Sm of <9 cm/s or < 10 cm/s, respectively, was associated with a threefold increased risk of all-cause mortality, underlining the prognostic value of the Sm measurements.

Our patients received 0.75 EMBs and/or angiographies per patient post OHT year. Patients who had been transplanted for at least 5 years (*n* = 77, mean 8.4 ±1.7 years post OHT) had a median of 3 (IQR 2–6) invasive diagnostic procedures during follow-up, which is an extremely low frequency compared to the guideline recommendations or to symptom-triggered biopsy protocols reported in the literature [4,15,16,17]. In 170 (35.4%) invasive procedures, EMBs or angiographies confirmed suspected AR or CAV, so that Sm-navigated invasive diagnostics can be referred to as a high-yield screening modality for post-OHT AR or CAV [18].

Our institutional conditional survival rates were comparable to the rates reported for de novo or post long-term circulatory support transplant recipients in large American databases or European transplant centers within the Eurotransplant or Scandiatransplant region [19,20,21,22].

LV-EF and strain analysis, as other echocardiographic parameters for LV myocardial function, have been described as predictors of graft failure after OHT. LV-EF decreases in advanced AR. But several pitfalls limit the accuracy of LV-EF measurements: (i) the endocardium border must be clearly visible in every segment of the apical 4- and 2-chamber view, (ii) apical foreshortening due to transducer angulation or atypical views leads to measurement errors, and (iii) the eye-balling technique for LV-EF estimation in cases of insufficient imaging quality is highly interobserver-dependent [23]. Due to these limitations, small changes in LV-EF, as reported for our patients with ISHLT1R AR, are not reliably detectable. Speckle-tracking-based global longitudinal strain (GLS) analyses and segmental longitudinal strain reliably indicate AR and CAV in OHT cohorts. Compared to TDI, this method is less angle- and interobserver-dependent. However, a major disadvantage is that high imaging quality is required; therefore, GLS is only applicable in selected patients [24,25,26]. 

Overall, we postulate clinical-Sm-triggered OHT follow-up care as a safe, noninvasive, reliable, and potentially cost-effective strategy for heart transplant recipients.

### Limitations

This analysis has certain limitations: The investigation was performed retrospectively in a single center and the samples of high-grade ACR and low-grade CAV were too small to perform statistical testing. Furthermore, we examined the role of echocardiographic surveillance in detecting histological rejection; the occurrence of symptoms and the need for treatment were not taken into account.

## Figures and Tables

**Table 1:** Baseline characteristics *n* = 166.

**Recipient**	
Age at OHT	46.2 (±11.4)
Sex (male)	127 (76.5)
BMI in kg/m²	25.2 (±4.0)
Waiting list status	
Highly urgent	146 (88.0)
Urgent (until February 2011)	4 (2.4)
Transplantable	16 (9.6)
Diagnosis leading to OHT	
DCM	114 (68.7)
HCM	4 (2.4)
RCM	0
ARVC	3 (1.8)
IHD	35 (21.1)
Other *	10 (6.0)
Previous VAD	84 (50.6)
LVAD	78 (47.0)
RVAD	1 (0.6)
BVAD	6 (3.6)
Concomitant disease	
CKD	115 (69.3)
DM	26 (15.7)
Type 1	4 (2.4)
Type 2	22 (13.3)
HLP	64 (38.6)
Former smoker	38 (22.9)
**Donor**	
Age at donation	40.8 (±14.1)
Sex (male)	107 (64.5)
BMI in kg/m²	25.2 (±3.7)
Echocardiography	
LV hypertrophy	12 (7.2)
Concomitant disease	
Hypertension	22 (13.3)
CAD	2 (1.2)
DM	6 (3.6)
Former smoker	26 (15.7)
Alcohol abuse	16 (9.6)
Drug abuse ^†^	7 (4.2)
Cardiopulmonary resuscitation	20 (12.0)
**Transplantation**	
Combined OHT	
Heart–kidney	3 (1.8)
Ischemic time in min	259.9 (±63.3)
ICU stay in days	26.7 (±36.3)
Hospital stay in days	62.3 (±50.7)
Inotropic support in days	8 (5–12)
Mechanical ventilation in days	5 (2–18)
GFR at discharge in ml/min	
>90	67 (40.2)
60–89	35 (21.1)
40–59	22 (13.3)
30–44	19 (11.4)
15–29	8 (4.8)
RRT	16 (9.6)
Immunosuppression induction	
Cyclosporine	162 (97.2)
Tacrolimus	4 (2.4)
Mycophenolate mofetil	9 (5.4)
Methylprednisolone	166 (100.0)
Antithymocyte globulin	100 (60.2)
Other ^§^	4 (2.4)
Immunosuppression at discharge	
Cyclosporine	92 (55.4)
Tacrolimus	71 (42.8)
Everolimus	19 (11.4)
Mycophenolate mofetil	132 (79.5)
Methylprednisolone	164 (98.8)
Unknown	2 (1.2)

ARVC, arrhythmogene right ventricular cardiomyopathy; BMI, body mass index; BVAD, biventricular assist device; CKD, chronic kidney disease; DCM, dilated cardiomyopathy; DM, diabetes mellitus; GFR, glomerular filtration rate; HCM, hypertrophic cardiomyopathy; HLP, hyperlipidemia; ICU, intensive care unit; IHD, ischemic heart disease; LVAD, left ventricular assist device; OHT, orthotopic heart transplantation; RCM, restrictive cardiomyopathy; RRT, renal replacement therapy; RVAD, right ventricular assist device; VAD, ventricular assist device; * others: transposition of the great arteries, noncompaction cardiomyopathy, peripartum cardiomyopathy; ^†^ drug abuse: unknown, cannabis, cocaine, polytoxicomania; ^§^ others: basiliximab, plasmapheresis, rituximab.

**Table 2:** Sm and LV-EF in CAV and ACR.

CAV Grade	ISHLT CAV1 *n* = 5			ISHLT CAV2 *n* = 4			ISHLT CAV3 *n* = 20			
	Pre	CAV1	Change	Pre	CAV2	Change	Pre	CAV3	Change	(CI) *p* value
Sm rad cm/s	10.7	9.9	−0.8	11.0	10.3	0.8	10.5	9.3	1.3	(0.23–2.43)
	(±1.2)	(±0.7)	(±1.69)	(±2.2)	(±1.5)	(±2.2)	(±1.9)	(±2.9)	(±2.5)	0.017
Sm long cm/s	11.0	9.2	−1.8	10.8	10.8	0	10.6	9.5	1.4	(0.21–2.66)
	(±1.4)	(±0.7)	(±1.3)	(±2.2)	(±2.2)	(±2.5)	(±2.2)	(±2.6)	(±2.8)	0.021
LV-EF %	60.0	56.0	−4.0	58.8	55.5	−3.75	56.9	52.1	4.8	(0.42–9.18)
	(±3.2)	(±11.6)	(±8.6)	(±4.1)	(±3.5)	(±1.1)	(±6.7)	(±12.0)	(±10.0)	0.031
	ACR grade			ISHLT 1R *n* = 84	ISHLT 2R *n* = 13			ISHLT 3R *n* = 8		
	pre	1R	change	(CI) *p* value	pre	2R	change	pre	3R	change
Sm rad cm/s	11.3	9.6	−1.7	(1.32–2.08)	11.3	9.1	−1.9	11.4	9.4	−2.6
	(±2.0)	(±2.0)	(±1.8)	<0.001	(±1.7)	(±2.4)	(±2.3)	(±2.1)	(±2.1)	(±3.0)
Sm long cm/s	11.8	10.0	−2.0	(1.66–2.34)	11.7	9.1	−2.3	11.9	9.1	−3.4
	(±1.7)	(±2.4)	(±1.6)	<0.001	(±1.8)	(±2.9)	(±2.4)	(±1.8)	(±1.8)	(±1.9)
LV-EF %	62.6	57.9	−5.3	(3.85–6.75)	60.4	53.8	−7.1	65.0	54.4	−10.6
	(±5.8)	(±8.8)	(±6.8)	<0.001	(±4.0)	(±12.7)	(±16.2)	(±3.5)	(±14.5)	(±13.3)

ACR, acute cellular rejection; CAV, cardiac allograft vasculopathy; CI, confidence interval; ISHLT, International Society for Heart and Lung Transplantation; long, longitudinal; LV-EF, left ventricular rejection fraction; rad radial; Sm, systolic wall motion peak velocity.

**Table 3:** Sm and LV-EF in AMR.

ISHLT AMR *n* = 65				
	Pre	AMR	Change (%)	(CI) *p* Value
Sm rad cm/s	11.7 (±1.9)	10.1 (±2.1)	−1.6 (±1.9)	(0.77–2.43) <0.001
Sm long cm/s	11.9 (±1.5)	10.3 (±2.0)	−1.8 (±2.0)	(0.92–2.67) <0.001
LV-EF %	60.8 (±6.4)	56.5 (±9.8)	−4.5 (±7.7)	(1.13–7.87) 0.009

AMR, antibody-mediated rejection; ISHLT, International Society For Heart And Lung Transplantation; long, longitudinal; LV-EF, left ventricular rejection fraction; rad radial; Sm, systolic wall motion peak velocity.

**Table 4:** Sm as a predictor of mortality.

	Hazard Ratio	95% CI	*p* Value
Sm long, cm/s	0.74	0.58–0.95	0.016
Sm rad, cm/s	0.77	0.63–0.93	0.008
IVSd per mm	0.94	0.72–1.21	0.607
LV-EF, %	1.0	0.95–1.05	0.968
Sm long ≥ 10 cm/s	0.34	0.14–0.84	0.019
Sm long < 10 cm/s	2.92	1.19–7.18	0.019
Sm rad ≥ 9 cm/s	0.31	0.11–0.83	0.020
Sm rad < 9 cm/s	3.24	1.2–8.76	0.020

CI, confidence interval; IVSd, interventricular septum enddiastolic; Sm, systolic wall motion peak velocity.
